# Social Self-Efficacy and Internet Gaming Disorder Among Chinese Undergraduates: The Mediating Role of Alexithymia and the Moderating Role of Empathy

**DOI:** 10.3389/fpsyg.2022.898554

**Published:** 2022-07-05

**Authors:** Yanhong Zhang, Ting Liang, Xiong Gan, Xueyan Zheng, Hao Li, Jingyue Zhang

**Affiliations:** Department of Psychology, College of Education and Sports Sciences, Yangtze University, Jingzhou, China

**Keywords:** social self-efficacy, internet gaming disorder, alexithymia, empathy, Chinese undergraduates

## Abstract

As an increasingly serious social problem, Internet gaming disorder (IGD) of college students may be related to their social self-efficacy. However, the relationship and its internal mechanisms underlying are still unclear. The current study tested the mediating effect of alexithymia in the association between social self-efficacy and IGD, and whether this mediating process was moderated by empathy. Social Self-Efficacy Scale (PSSE), Chinese version of Internet Game Addiction Scale (IGAS-C), Toronto Alexithymia Scale (TAS-20) and Interpersonal Response Scale (IRI-C) were adopted to examine the model on 888 Chinese college students. The results indicated that social self-efficacy was significantly negatively associate with IGD. Mediation analysis illustrated that alexithymia mediated the association between social self-efficacy and IGD. Further, moderated mediation analysis manifested that the mediated effects was stronger for lower level of empathy. The conclusions corroborate and clarify the mechanisms that alexithymia mediated the association between social self-efficacy and IGD, and the mediation effects is moderated *via* empathy. Besides, these findings provide available references for colleges to conduct educational activities, and at the same time provide scientific suggestions for preventing IGD among undergraduates.

## Introduction

With the rapid development of society and technology, the internet has become an imperative component of our daily life. According to the 48th Statistical Reports on Internet Development in China, as of June 2021, the number of internet users in China had reached 1.011 billion, while the number of online game users had reached 510 million, including more than 100 million young users (China Internet Information Center, 2021). Along with this trend, the number of internet gaming disorders is rising swiftly. Internet gaming disorder (IGD) is an intense and sustained addictive behavior of compulsive desires and dependences to enjoy the computer or video games. Harmful withdrawal reaction occurs while the addict cease using it, which will result in the obvious impairment of social and psychological functions of addicts (Moudiab and Spada, 2019). IGD is characterized by excessive attention, obsessiveness, lack of control, and impulsive behavior (Tateno et al., 2019). As a behavioral addiction, IGD has been found to bring about various levels of psychological problems, such as somatization, interpersonal sensitivity (Mannikko et al., 2020), anxiety and depression (Ostovar et al., 2016). Previous studies reported that the prevalence of IGD varied from 1.2 to 46% (Lemmens et al., 2015; Shi et al., 2020). As the main group of internet users, college students are also a high incidence group of IGD. Therefore, the factors and potential mechanisms which affect the development of IGD are required to identified, and the effective evidence-based interventions are urgent developed to prevent IGD among college students.

Among the many factors associated with IGD, self-efficacy has attracted much attention of researchers (Chen et al., 2020). As the sense of self-efficacy in the field of social communication, social self-efficacy is a special kind of self-efficacy (Bandura, 1997). Social self-efficacy, one aspect of effective social skills, refers to a readiness to initiate behavior in social conditions and the individuals’ beliefs that they are able to initiate social contact and develop new friendships (Smith and Betz, 2000). According to the social control theory, individuals who have less contact with important social groups (i.e., schools and peers) will be less restrained and influenced by these groups, thereby increasing risky behaviors (i.e., addiction and crime) (Church et al., 2012). Generally, social adaptation has protective effects on all aspects of core self-evaluation, thus individuals with high evaluation of self-social competence will not devote extensive energy to online games (Geng et al., 2018). Previous studies have shown that a high level of self-efficacy is related to high levels of self-appreciation and low levels of depression and social anxiety (Xie et al., 2020). Others have shown that low levels of social self-efficacy are related to a high level of internet addiction as well as games addiction (Gazo et al., 2020). Consequently, the present study investigates the relationship between social self-efficacy and IGD, and puts forwards the hypothesis that social self-efficacy can negatively correlate with IGD (H1). Simultaneously, the research can provide a theoretical basis for colleges and universities to prevent students from IGD.

### The Mediating Role of Alexithymia

It is important, therefore, to explore the potential mediators of social self-efficacy and their possible effects on IGD. From this perspective, alexithymia can be a useful frame for explaining the possible relationship between social self-efficacy and IGD. Alexithymia describes an inability to articulate and interpret internal feelings (Taylor, 2000). Its central characteristics were poor capabilities to acknowledge and verbalize feelings, lack of fantasy, and propensity for practical action-oriented thinking (Hao et al., 2019). Alexithymia has been proved to be a personality trait by growing evidences (Salminen et al., 2006). According to self-determination theory, individuals usually have relationship demands, autonomy demands and ability demands (Edward et al., 2001). Individuals with low self-efficacy often show inferiority, helplessness and avoidance (Aune et al., 2021). They avoid frequent communication with other, thus their basic demands are difficult to satisfied (Lau et al., 2018; Bozzato, 2020). In such an indifferent and depressed environment, individuals are more likely to suffer from alexithymia. Recent studies find that social self-efficacy displayed significant connection with alexithymia (Mallinckrodt and Wei, 2005). For one thing, individuals with low social self-efficacy are reluctant to actively interact with others to exchange ideas, explore novelties, and rarely express their emotional needs (Naeem et al., 2015; Slanbekova et al., 2019). Furthermore, they are prone to lack of imagination and outward thinking, which are the significant characteristics of alexithymia (Chung et al., 2016). For another, the difficulty of emotion recognition and processing are also the main symptom of alexithymia (Velotti et al., 2019). As a social interaction behavior, the recognition, expression and control of emotions are inevitably affected by the individual’s social cognitive level (De Guzman et al., 2016). In general, individuals with high self-efficacy have stronger ability to recognize and regulate emotions. As a result, the social self-efficacy of college students may have a certain impact on their alexithymia level.

Alexithymia can be related to IGD (Bonnaire and Baptista, 2019). Many researchers have indicated that alexithymic individuals who were weak in emotion regulation skills tend to adopt compulsive behaviors and addictive behaviors to compensate (Taylor et al., 1991), such as pathological gambling, substance using disorders, and eating disorders (Barth, 2015). Due to the deficit cognitive capabilities, alexithymic individuals usually have difficulties in facing and dealing with stressful conditions, thus they are more likely to suffer from negative emotions like anxiety, depression and stress (Kishon et al., 2019). As a result, individuals often struggle to find their life purpose and meaning, eventually falling into boredom and turning their attention to online games (Eastwood et al., 2007). More importantly, alexithymia often exhibits an extroverted cognitive style and is unable to experience their emotions directly or deeply. Therefore, they tend to perceive that external stimuli are monotonous and lack vitality, experience more sense of loss and meaninglessness, which ultimately leads to online gaming addiction (Zhang Y. L. et al., 2020). By and large, it is reasonable to assume that social self-efficacy can trigger alexithymia, which in turn will bring about IGD. In other word, alexithymia can mediate the relation between social self-efficacy and IGD. On the basis of the theoretical grounds and empirical evidence, we proposed the Hypothesis as follow: Alexithymia would partially mediate the relation between social self-efficacy and IGD (H2).

### The Moderating Role of Empathy

Despite social self-efficacy can affect alexithymia, not all individuals are influenced by social self-efficacy and suffer from alexithymia equally. The reason may be that there are some other individual internal factors that probably moderate the association between social self-efficacy and alexithymia, for example, empathy. Empathy entails the ability to perceive, understand and feel the emotional states of others (Eisenberg and Paul, 1987). Two components of empathy have been distinguished: cognitive empathy refers to the ability to understand others’ emotions, while affective empathy refers to the ability to appraise an individual’s emotional responses to others’ emotions (Davis, 1983). Empirical studies have confirmed that the level and content of empathy may affect individuals’ emotional state. For instance, Knight et al. (2019) found that individuals’ empathy for painful emotion can positively predict anxiety. However, Morelli et al. (2015) hold that empathy positively predicted the tendency of social relationship and simultaneously negatively predicted the perceived loneliness. Emotional state has an impact on mobilizing cognitive resources to identify and express oneself (Yan and Su, 2021). Therefore, empathy may be a protective factor of the relationship between social self-efficacy and alexithymia. According to the self to other model of empathy (SOME), individuals who have certain problems in the self/other feeling transitions are less likely to have capacity for empathy generation and expression, and may in turn lead to behavioral problems (such as anxiety disorder, conduct disorder, and alexithymia) (Bird and Viding, 2014). People having higher degrees of empathy narrow the gap between self-evaluation and evaluation of others, and may have greater degree of overlaps with others in terms of neural and mental representations (Preston and Hofelich, 2012). They are more likely to feel kindness from others, receive more social support, and are willing to try to express themselves and show their emotions even when they perceive themselves as socially incompetent (Galinsky and Ku, 2004). On the contrary, individuals who lack empathy are usually more self-centered and experience less social support, trust, and understanding (Lin et al., 2022). Therefore they are less disclosure of their emotions and feelings, and more likely to suffer from alexithymia. To sum up, it is reasonable to consider that empathy may alleviate the associations between social self-efficacy and alexithymia. Hypothesis 3: The relationships between social self-efficacy and alexithymia will vary with empathy. Concretely, the associations between social self-efficacy and alexithymia are stronger among individuals with lower empathy.

### The Present Study

**FIGURE 1:**
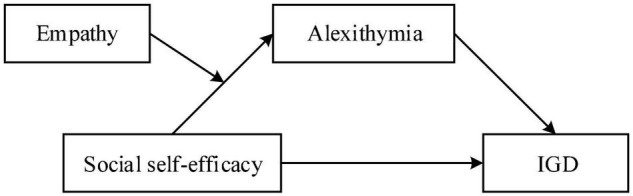
The proposed moderated mediation model.

The effect of social self-efficacy on IGD, and the potential mediating mechanism (i.e., how social self-efficacy is related to IGD) remain unclear. Furthermore, the function of moderating variables which probably touch upon the association is still unclear to a great extent (i.e., when the relationship between social self-efficacy and IGD is most potent). Taken together, the present study explored whether alexithymia would mediate the associations between social self-efficacy and IGD, and whether empathy would act as a moderator between social self-efficacy and alexithymia (Figure 1).

## Materials and Methods

### Participants and Procedures

The present study was approved by the Institutional Review Board of Yangtze University. Random sampling was adopted to conducted online testing on the students from various colleges and universities in Hubei province. After obtaining informed consent from the participants, the research was conducted by trained senior students majoring in psychology. All participants were told that their answers would be protected and that they were free to withdraw from the study at any time. A total of 1,000 students completed all the questionnaires, and 888 valid questionnaires remained after excluding invalid ones. The age of the subjects was between 17 and 25 years old (*M* = 21.08, *SD* = 1.57). Among them were 399 males (44.9%), 489 females (55.1%); The participants included 100 freshmen (11.3%), 253, sophomores (28.5%), 214 juniors (24.1%), 304 seniors (34.2%), and 17 students in fifth college graders (1.9%). Furthermore, the average Internet age was 7.99 years (*SD* = 3.372), and time spending on online game per day was 2.53 h (*SD* = 2.369).

### Measures

#### Social Self-Efficacy

The Social Self-Efficacy Scale (PSSE) was revised by Meng et al. (2012) based on the initial version compiled by Smith and Betz (2000). The revised version consists of 18 items, and it assesses a single factor. All the items were rated on a five-point scale (1 = very lack of confidence, 5 = great confidence). In this scale, higher scores mean great social self-efficacy. The scale has good reliability and validity in many studies (Zhang X. et al., 2020). The Cronbach’s α was 0.943.

#### Internet Gaming Disorder

IGD was assessed by the Chinese version of Internet Game Addiction Scale for college students (IGAS-C) (Liu et al., 2007). The questionnaire consisted of 24 items and measured IGD from five sources, including time management, mood experience, life conflict, sacrifice social, and withdrawal difficulty. All the items were rated on a five-point Likert-type scale (1 = “Not at all,” 5 = “Extremely”). Higher total scores reflect that the participant has higher level of IGD. This measure has demonstrated good reliability and validity among Chinese college students (Liang et al., 2020). Cronbach’s alpha for this scale was 0.969.

#### Alexithymia

Toronto Alexithymia Scale-20 (TAS) was used to measure individual’s alexithymia level (Bagby et al., 1994). Its Chinese version has been widely used and proved to have good validity for Chinese participants (Han et al., 2012). It includes 20 items and is based on a 5-point Likert scale ranging from “does not describe me well” (1) to “describes me very well” (5). The higher score indicates the participant has a higher level of alexithymia. The Cronbach’s alpha was 0.876.

#### Empathy

Empathy was assessed using the Interpersonal Response Indicator Scale (IRI-C), which was developed by Davis and inspected with great reliability and validity (Davis, 1980). The scale includes 22 items, and encompasses four subscales: Personal Distress, Perspective Taking, Fantasy, and Empathic Concern. Especially, all the items were presented in the form of a five-point scale (1 = Strongly disagree, 5 = Strongly agree). In this scale, higher total scores suggested that participants had higher level of empathy. This measure has demonstrated good reliability and validity among Chinese college students (Yang et al., 2020). Cronbach’s α for the empathy was 0.866.

#### Control Variables

In the proposed model, gender, grade, and time spending on online game per day are regarded as the control variables, as they have been found to be closely associated with our main variables in existing researches (Ding et al., 2018; Balhara et al., 2020).

### Statistical Analyses

Before statistical analysis, responses with the missing data were excluded from the data processing since the proportions of missing data for all variables were low (<1%). Firstly, descriptive statistics, the analyses of variance and correlation were conducted with IBM SPSS 22.0. Secondly, Hayes’ PROCESS macro (Model 4) (Hayes, 2013) was performed to test the mediating role of alexithymia in the association between social self-efficacy and IGD. Finally, whether the mediation process was moderated by empathy was further explored with Hayes’ PROCESS macro (Model 7) (Hayes, 2013). Furthermore, simple slopes analyses were performed to decompose all the potential significant interaction effects. In order to acquire robust standard errors for parameter estimation, the bootstrapping approach was introduced to measure the significance of the effects (Hayes, 2013). The bootstrapping method produced 95% bias-corrected confidence intervals of these effects from 1,000 resamples of the data. Confidence intervals that do not include zero mean effects are significant.

## Results

### Common Method Deviation Test

The Harman one-factor test was used to test for common method bias. The results showed 12 factors with characteristic roots greater than 1, explaining the amount of variance explained by the first factor was 21.95%, when the critical criterion was under 40%, indicating no serious common method bias variance.

### Primary Analysis

The mean, standard deviation, and correlation coefficient matrices for all variables are presented in Table 1. As hypothesized, social self-efficacy was negatively correlated with IGD and alexithymia, but positively correlated with the empathy. Alexithymia was positively correlated with IGD, but negatively correlated with the empathy. And, empathy was positively correlated with IGD. Furthermore, gender was negatively correlated with IGD. Grade was negatively correlated with both alexithymia and IGD. Time spending on online game per day was positively correlated with both alexithymia and IGD, and negatively correlated with both social self-efficacy and empathy. Given that social self-efficacy was negatively correlated with IGD, Hypothesis 1 was supported.

**TABLE 1:** Means, standard deviations and correlation coefficients of each variable.

Variables	*M*	*SD*	1	2	3	4	5	6	7
1 Gender	–	–	1						
2 Grade	–	–	0.34**	1					
3 Time spending on online game per day	2.53	2.37	−0.11**	−0.03	1				
4 Social self-efficacy	3.20	0.74	−0.34	0.07	−0.14**	1			
5 Alexithymia	2.93	0.47	−0.05	−0.07**	0.17**	−0.43**	1		
6 Empathy	2.58	0.47	0.02	0.02	−0.1**	0.24**	−0.43**	1	
7 IGD	2.57	0.94	−0.27**	−0.13**	0.43**	−0.30**	0.35**	−0.21**	1

*P < 0.05, **P < 0.01, ***P < 0.001.

### Testing for the Mediating Role of Alexithymia

**FIGURE 2:**
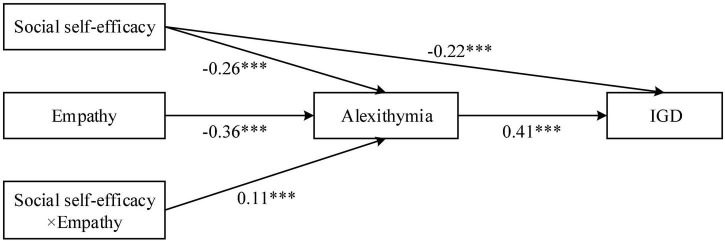
Moderated mediation analysis. *p < 0.05, **p < 0.01, ***p < 0.001.

In accordance with the anticipation of Hypothesis 2, alexithymia played a mediating role in the relationship between social self-efficacy and IGD. Model 4 of PROCESS macro (Hayes, 2013) was performed to test this hypothesis. As illustrated in Table 2, social self-efficacy significantly negatively predicted IGD (*β* = −0.33, *p* < 0.001). In addition, results simultaneously indicated social self-efficacy significantly negatively predicted alexithymia (*β* = −0.26, *p* < 0.001). As it can be seen in Figure 2 and Table 2, alexithymia is prominently and positively predicted online game addiction (*β* = 0.41, *p* < 0.001). Further, the bias-corrected percentile bootstrap method indicated that the indirect effect social self-efficacy on IGD *via* alexithymia was significantly (*β* = −0.22, *p* < 0.001), *ab* = −0.11, 95%, *CI* = [−0.15, −0.07]. In addition, the mediation effect accounted for 33.3% of the total effect. The above results provided compelling evidence that social self-efficacy was associated with the increase of IGD, moreover, the relation was mediated by alexithymia simultaneously. Consequently, Hypothesis 2 was fully verified.

**TABLE 2:** Regression analysis of the mediation effects of alexithymia.

Regression equation		Overall fitting index			Regression coefficient	
Variables	Predictors	*R*	*R* ^2^	*F*(*df*)	*β*	*t*
IGD	Gender	0.54	0.30	92.83***(4)	−0.43	−7.41**
	Grade				−0.02	−0.77
	Online time				0.15	12.78**
	Social self-efficacy				−0.33	−0.89***
Alexithymia	Gender	0.45	0.20	54.82***(4)	−0.04	−1.22
	Grade				−0.01	−0.71
	Online time				0.02	3.66***
	Social self-efficacy				−0.26	13.49***
IGD	Gender	0.57	0.33	86.40***(4)	0.41	−7.31***
	Grade				−0.02	−0.63
	Online time				0.14	12.18***
	Social self-efficacy				−0.22	−5.62***
	Alexithymia				0.41	6.55**

*p < 0.05, **p < 0.01, ***p < 0.001.

### Testing for Moderated Mediation

**FIGURE 3:**
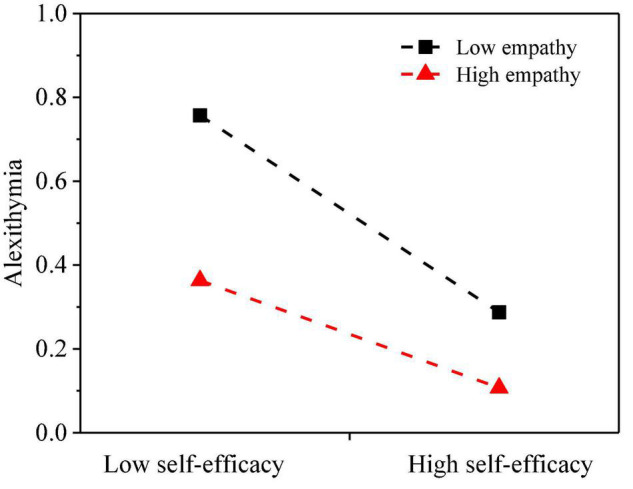
Empathy moderates the link between social self-efficacy and alexithymia.

Hypothesis 3 assumed that the association between social self-efficacy and alexithymia will vary with empathy. To examine the hypothesis, Model 7 of PROCESS macro (Hayes, 2013) was performed. The main results of the moderated mediation analysis are presented in Figure 2 and Table 3. The effect of social self-efficacy on alexithymia was significant (*β* = −0.22, *p* < 0.001); and more importantly, the effect was moderated by empathy (*β* = 0.11, *p* < 0.001). Additionally, simple slope analyses were conducted to illustrate this significant interaction and explore whether slopes for the high-empathy group (1 *SD* above the mean) were different from slopes for the low-empathy group (1 *SD* below the mean) in the mediator variable model. The results were plotted in Figure 3. The results of simple slope tests shown that the association between social self-efficacy and alexithymia among individuals low in empathy was relatively stronger (*β* simple = −0.25, *p* < 0.01) than those who high in empathy (*β* simple = −0.15, *p* < 0.01). Therefore, Hypothesis 3 was supported.

**TABLE 3:** Regression analysis of the moderated effects of empathy.

Regression equation		Overall fitting index			Regression coefficient	
Variables	Predictors	*R*	*R* ^2^	*F*(*df*)	*β*	*t*
Alexithymia	Gender	0.56	0.32	67.74***(6)	−0.03	−0.90
	Grade				−0.01	−0.73
	Online time				0.02	3.00***
	Social self-efficacy				−0.20	−10.45***
	Empathy				−0.36	−12.25***
	Social self-efficacy × Empathy				0.11	3.45***

*p < 0.05, **p < 0.01, ***p < 0.001.

## Discussion

Based on social control theory (Church et al., 2012), self-determination theory (Edward et al., 2001), and the self to other model of Empathy (Bird and Viding, 2014), a moderated mediation model was formulated to examine whether social self-efficacy would be indirectly related to IGD *via* increased alexithymia and whether this indirect association was moderated by empathy. These findings facilitate our understanding of “how” and “for whom” social self-efficacy impacts IGD, and they also provide insights into the intervention of behaviors of IGD among undergraduates.

### Social Self-Efficacy and Undergraduates Internet Gaming Disorder

The current study showed that social self-efficacy was negatively associated with IGD. Prior studies have identified social anxiety as a potential influencing factor among IGD in undergraduates (Nasution et al., 2019). The current study adds to this line of literature and reveals that social self-efficacy is also related to IGD. Our result is in line with the previous research results (Verrastro et al., 2021). According to the cognitive-behavior model, a variety of maladaptive cognition might cause problem behaviors such as IGD (Davis, 2001). Moreover, Individuals with lower social self-efficacy generally fear to talk with strangers and avoid any social situations, which is one of the important manifestations of maladaptive cognition (Gong et al., 2020). Thus, Individuals with lower social self-efficacy may increase the risk of IGD. Meanwhile, internet games have the characteristics of anonymity, low pressure and competitiveness, which let individuals with low social self-efficacy meet their unmet social needs and to obtain physiological and psychological satisfaction (Wei et al., 2005). In line with this view, it has been shown that gamers may tend to avoid the inadequacy felt in the offline world by creating a different and more confident online social self (Carlisle et al., 2019).

### The Mediating Role of Alexithymia

This study not only focuses on the direct impact of social self-efficacy on IGD, but also pays attention to the indirect impact of alexithymia on IGD. As stated in Hypothesis 2, the results demonstrate that social self-efficacy can directly and negatively predict IGD among undergraduates, and it can also indirectly predict IGD through alexithymia, which is consistent with the previous research results (Mahapatra and Sharma, 2018). In reality, individuals with low social self-efficacy lack confidence in self-social abilities. They are often exposed to problems such as social adaptation difficulties and interpersonal isolation, which will lead to a decline in the individual’s ability to recognize and describe the emotional state of others or themselves (characteristics of alexithymia) (Paricio et al., 2020). Individuals with high-level alexithymia have certain deviations in the selection of information and the recognition of emotional schemas, thus have no capability to recognize facial expressions effectively, which will eventually lead to communication anxiety (Radetzki et al., 2021). More importantly, the convenience and evasiveness of the internet allow the anxious individuals to dodge the stressful behaviors caused by face-to-face communication (Chu et al., 2021). They don’t need to identify other people’s emotions in real time, let alone express their emotions accurately and timely. Sometimes a single emoticon can complete an interpersonal communication. Even in online games, a skill or scene can express their ideas as well (Xue et al., 2021). As a result, the less stressful online setting makes it a more secure and comfortable socialization option for individuals who are socially anxious (Valkenburg and Peter, 2009). In addition, the results in this study are also consistent with the prediction of the compensation theory of internet addiction (Kraut et al., 2002). That is, internet gaming addictive behavior is a psychological compensation behavior adopted by individuals when they suffer from mental distress, so social self-efficacy can negatively predict undergraduates’ IGD.

### The Moderating Role of Empathy

As envisaged in Hypothesis 3, this study indicated that empathy plays a moderating role between social self-efficacy and alexithymia. In particular, the social self-efficacy of individuals with low empathy has a greater negative impact on alexithymia than that of individuals with high empathy. These findings are congruent with the theoretical deduction from the self to other model of empathy (SOME) and also consistent with previous research (Bird and Viding, 2014). On the one hand, some previous studies have shown that emotional empathy is significantly negatively correlated with social anxiety (Morrison et al., 2019). Emotional empathy is manifested as the ability to share the emotions of others, which has a positive impact on interpersonal relationships. In other words, for the individuals with lower-level of emotional empathy, tense interpersonal situations will increase negative emotions (such as anxiety, distress), and ultimately lead to alexithymia. On the other hand, there is some evidence that cognitive empathy has a positive impact on individual disclosure (Ibrahimoglu et al., 2021). The understanding of other people’s emotions and psychology will affect the interpretation of stimuli and the cognitive construction (Brett and Maybery, 2021). Thus, individuals with low empathy frequently reduce their desire to express or interpret internal feelings.

### Limitations and Future Directions

The following limitations need to be noted before interpreting the results of our study. Firstly, its cross-sectional and correlational nature does not allow for causal interpretations of the data. Therefore, longitudinal research may be a better option for future research. Secondly, our study employed the self-report approach, thus there might be more or less biases in participants’ respond, which probably impacts the authenticity of results and the verifiability of the data (Podsakoff et al., 2012). Besides, more comprehensive data from various informants should be collected in combination with a variety of other methods if conditions permit. Thirdly, it is still unknown whether our research results on the basis of convenience sample of undergraduates are able to be extended to clinical samples. Finally, the present study chose to focus intensively on internal factors, but actually we are aware that many external or environmental factors could also have certain impacts (Lin et al., 2020). Utilizing multiple data sources, concurrently examining individual and environmental factors probably can advance insight into critical determinants of IGD.

### Practical Implications

In spite of the above limitations in our study, there are still certain theoretical and practical implications. First of all, given that social self-efficacy is negatively associated with the IGD among undergraduates, universities should provide courses which taught college students to improve skills for recognizing maladaptive cognitions during social interactions and effective interpersonal communication, thereby enhancing the students’ self-confidence and reducing social anxiety. Secondly, our study found that alexithymia mediated the association between social self-efficacy and IGD, which indicates alexithymia plays a significant role in increasing undergraduates’ IGD. The above findings remind us it might have some benefits for college students to strengthen their ability of expressing inner thoughts to a certain extent. Additionally, at the theoretical aspect, our findings have certain significance for deepening the comprehending of the influencing factors of IGD among college students. Thirdly, the relation between social self-efficacy and alexithymia is stronger for undergraduates with a lower level of empathy. Due to the essential exacerbating role of empathy, increasing undergraduates’ empathy could serve as an indirect intervention strategy for decreasing IGD.

## Conclusion

To sum up, our study found that social self-efficacy was positive associated to IGD. Moreover, alexithymia was proved to mediate the association between social self-efficacy and IGD in the mediation analysis. Simultaneously, the moderated mediation analysis reveals that empathy would exacerbate the relation between social self-efficacy and alexithymia. Concretely, the association between social self-efficacy and alexithymia would be stronger for undergraduates with a lower level of empathy.

## Data Availability Statement

The raw data supporting the conclusions of this article will be made available by the authors, without undue reservation.

## Ethics Statement

The studies involving human participants were reviewed and approved by the Research Ethics Committee of College of Education and Sports Sciences, Yangtze University. Written informed consent to participate in this study was provided by the participants or their legal guardian/next of kin.

## Author Contributions

YZ, TL, XG, and XZ designed the work and were responsible for the overall development of this study, including the planning of sample collection, data analysis, writing, and polishing of the manuscript. TL, HL, and JZ were in charge of data collection and analysis of this study. YZ, TL, and XG were in charge of the main revision for this manuscript. YZ and TL were responsible for revising the manuscript and made a great contribution to the final acceptance of the manuscript. YZ provided the manuscript fee. All authors contributed to the article and approved the submitted version.

## Conflict of Interest

The authors declare that the research was conducted in the absence of any commercial or financial relationships that could be construed as a potential conflict of interest.

## Publisher’s Note

All claims expressed in this article are solely those of the authors and do not necessarily represent those of their affiliated organizations, or those of the publisher, the editors and the reviewers. Any product that may be evaluated in this article, or claim that may be made by its manufacturer, is not guaranteed or endorsed by the publisher.
